# Convergence of Phenomenology and Neuroscience in Autism Therapy: A Case Study of Exchange and Development Therapy

**DOI:** 10.1192/j.eurpsy.2025.587

**Published:** 2025-08-26

**Authors:** S. Bora

**Affiliations:** 1 Tours Regional University Hospital, Tours, France; 2 University of Central Lancashire, Preston, United Kingdom; 3 Cambodian Children’s Fund, Phnom Penh, Cambodia

## Abstract

**Introduction:**

This abstract explores the integration of phenomenology and neuroscience in understanding and managing Autism Spectrum Disorder (ASD) through Exchange and Development Therapy (EDT). EDT, developed by Professor Gilbert Lelord, focuses on restoring impaired functions in children with autism through principles of calm, receptiveness, and reciprocity.

**Objectives:**

This study investigates the similarities between EDT principles and phenomenological concepts like intersubjectivity, intercorporeality, and intentionality, aiming to demonstrate the value of phenomenology in understanding autism.

**Methods:**

The research involved a two-part approach:

1. Theoretical argumentation, explaining EDT principles and relevant phenomenological concepts in autism.
2. Phenomenological analysis of autobiographies of individuals with autism to identify recurring themes and align them with EDT principles.

**Results:**

The study revealed a significant convergence between EDT principles and phenomenological concepts. Calmness in EDT aligns with intercorporeality, receptiveness with intentionality, and reciprocity with intersubjectivity. This convergence suggests that phenomenology is crucial for understanding the lived experiences of individuals with autism, which can inform and enhance neuroscience-based therapeutic approaches.

**Conclusions:**

The integration of phenomenology and neuroscience offers a promising avenue for developing more effective and compassionate autism therapies. By incorporating the subjective experiences of individuals with autism, therapists can tailor interventions that resonate with their unique phenomenological worlds, leading to improved therapeutic outcomes. This research advocates for a more holistic and individualized approach to autism treatment, highlighting the indispensable role of phenomenology in contemporary psychiatry.

**Disclosure of Interest:**

S. Bora Grant / Research support from: International Exchange Award from the Renewing Phenomenological Psychopathology funded by the Wellcome Trust

